# Effect of ginger on the blood glucose level of women with gestational diabetes mellitus (GDM) with impaired glucose tolerance test (GTT): a randomized double-blind placebo-controlled trial

**DOI:** 10.1186/s12906-020-02908-5

**Published:** 2020-04-19

**Authors:** Fariba Hajimoosayi, Shahideh Jahanian Sadatmahalleh, Anoshirvan Kazemnejad, Reihane Pirjani

**Affiliations:** 1 Department of Midwifery and Reproductive Health, Faculty of Medical Sciences, Tarbiat Modares University, P.O. Box: 1415-111, Tehran, Iran; 2 Department of Biostatistics, Faculty of Medical Sciences, Tarbiat Modares University, Tehran, Iran; 3 Department of Obstetrics and Gynecology, Arash Hospital, Tehran University of Medical Sciences, Tehran, Iran

**Keywords:** Ginger, Gestational diabetes mellitus, Insulin, BS2hpp

## Abstract

**Background:**

Gestational diabetes mellitus (GDM) is one the most common complications of pregnancy. The present work aimed at investigating the effect of ginger on the blood glucose level of GDM women with impaired glucose tolerance test (GTT).

**Methods:**

This randomized double-blind placebo-controlled clinical trial was performed on the total of 70 women with GDM, who were in 24–28 weeks of pregnancy with impaired GTT from 2015 to 2016. For this purpose, the women were assigned to two groups of ginger or placebo. The ginger group received 126 tablets of ginger, and the placebo group received 126 tablets of placebo for six weeks. The serum Blood Sugar 2 h post-prandial (BS2hpp), Fast Blood Sugar (FBS) and insulin, as well as Homeostasis Model Assessment (HOMA) index were analyzed before and six weeks after intervention.

**Results:**

The mean of FBS (*P* = 0.04), fasting insulin (*P* = 0.01), and HOMA index (*P* = 0.05) was reduced significantly in the ginger group six weeks after intervention in comparison to the placebo group. But the mean of BS2hpp did not show any significant reduction in the two groups (*P* > 0.05(.

**Conclusions:**

Oral administration of ginger tablet improved FBS, serum insulin and HOMA index in the women with GDM; however, it could not reduce their BS2hpp level.

The trial has been registered in the Iranian Registry of Clinical Trials (IRCT2015090523897N1).

## Background

Impaired glucose tolerance occurring for the first time during pregnancy is called Gestational Diabetes Mellitus (GDM). GDM prevalence of 1–14% has been reported in different studies [1]. Risk factors in GDM include high maternal age and weight before pregnancy, previous GDM, glycosuria in the current pregnancy, familial history of Diabetes Mellitus (DM) and born of a macrosomic baby [2]. Some studies have shown that GDM is associated with several perinatal complications such as polyhydramnios, macrosomia, hypoxemia, hypocalcemia in the fetus, gestational hypertension, preeclampsia and elevated risk of cesarean section in the mother [2–4]. Today many herbal drugs are used to treat the diseases throughout the world. Ginger is one of the widely used spices and functional foods. The medicinal features attributed to ginger are anti-arthritic, anti-migraine, anti-thrombotic, anti-inflammatory, anti-nausea, anti-vomiting, hypolipidemic and analgesic properties [5, 6].

Also it has been reported that ginger is a hypoglycemic food adjunct in both animals and humans [6–8] In recent years, ginger has been used for the treatment of Type 2 Diabetes (T2D) in human subjects [9].

Several other mechanisms can explain the beneficial effects of ginger on improving glucose level of pregnant women with GDM. For example, some studies mention that ginger inhibits enzymes involved in carbohydrate metabolism. The key enzymes controlling carbohydrate metabolism associated with hyperglycaemia and T2D are ɑ-amylase and ɑ-glucosidase. The ethyl acetate extract of ginger has the highest ɑ-glucosidase and amylase inhibitory activity, so it may perfectly control the glucose statuse.

Ginger increases insulin release and sensivity. Recent studies have shown that ginger can modulate insulin release. This herbal medicine promotes glucose clearance in insulin responsive peripheral tissues, which is crucial in maintaining blood glucose homeostasis. Findings of in vitro studies have suggest that ginger extract and its pungent gingerol principles enhance glucose uptake. Ali Taghizade Afshari in a clinical trial demonstrated that ginger causes an increase in the anti-oxidant capacity of plasma and reduces renal nephropathy in diabethic rats, means the positive effect of ginger on diabetes [10]. Farzad Shidfar et al. also conducted a double blinded placebo-controlled randomized clinical trial on two groups of patients with Type 2 Diabetes Mellitus (T2MD) to test the effect of ginger powder on them. After three months of treatment of the patients with 3 g of ginger powder or placebo, they reported an improvemrnt of glycemic indices as well as TAC and PON-1 activity in the patients [7]. Ganiyu Oboh et al. in a clinical trial compared the phenolic contents and anti-oxisdant properties of two types of ginger (white and red) and their inhibitory effects on the activity of enzymes linked to T2D using an in vitro model. The results showed that both varieties of ginger exhibited mild ɑ-amylase and stronger ɑ-glucosidase inhibitory activities, demonstrating that they can be potentially used in management or control of postprandial hyperglycemia associated with T2MD [11]. Sanjay P. Akhani et al. tested the effect of ginger juice for six weeks on streptozotocin-induced type 1 diabetic rats. They reported that treatment of rats with ginger resulted in an increase in insulin levels and a decrease in fasting glucose levels in the diabetic rats. In addition, ginger caused a decrease in serum cholesterol, serum triglyceride and blood pressure in the studied rats [12]. These studies suggest the potential antidiabetic activity of ginger in hyperglycemic statues. If GDM stays unchecked, it would have maternal and fetal complications, and if controlled, pregnancy outcome will be better. Diet therapy, life style changing and physical activity are some of treatments of GDM but pregnant women can not change their style easily. For example, weight loss has limitation for them. On the other hand, oral antidiabetic drugs are not routinely used yet. At last, those pregnant women whose blood glucose level is not controlled with these types of treatment, must begin insulin therapy. So we were interested to study about the effect of ginger on blood glucose.

This randomized double-blinded clinical trial was conducted to evaluate the efficacy of ginger on the blood glucose level of women with GDM with impaired glucose tolerance test (GTT).

## Methods

**Fig. 1:**
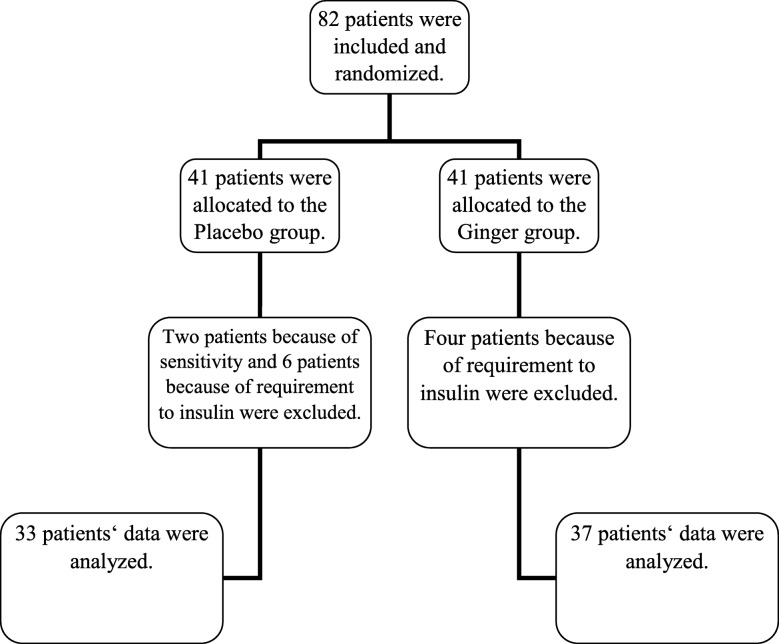
Studied patients ʼ flow diagram

This randomized, double-blind placebo-controlled study was performed on GDM women with impaired GTT at their 24–28 weeks of pregnancy at the Prenatal Section of Arash Hospital in Tehran, Iran from April 2015 to April 2016. Based on the results of the study of Arabloo [9], who compared the level of blood biochemical variables pre- and post-intervention between the two study groups, with 99% confidence interval and 90% power of test, the sample size was determined as 29 individuals for each arm, and by counting 30% sample losing, the minimum sample size was estimated 38 individuals in each group that means totally 76 pregnant women with GDM. At last, 82 consecutive women with GDM (41 persons in each group) who had the inclusion criteria were recruited for the present study (Fig. 1).

More recently, the International Association of the Diabetes in Pregnancy Study Group (IADPSG), after extensive analysis of the Hyperglycemia and Adverse Pregnancy Outcomes (HAPO) study, recommended new diagnostic criteria for GDM [13] based on the two hour 75 g OGTT: a fasting glucose ≥5.1 mmol/L (92 mg/dl), or a one hour result of ≥10.0 mmol/L (180 mg/dl), or a two hour result of ≥8.5 mmol/L (153 mg/dl).

If the Fast Blood Sugar (FBS) level was higher than 92 mg/dl, first hour post-prandial blood glucose level was higher than 180 mg/dl and 2 h post-prandial was higher than 153 mg/dl, pregnant women were considered to have GDM According to a computer-generated list of random number groups, each woman was randomly allocated to each arm. All participants as well as the attending doctor were blind to the ginger and placebo tablets and the active drug. The investigator was not involved in the recruitment of women, gave the tablets to the participants only based on the computer-generated randomization list, and assigned the treatment group according to the sequence of the recruitment. The codes were broken at the end of study. Before the enrollment, all women gave a written informed consent to participate in the study. The investigator explained the nature, purpose and risks of the trial and provided them with a copy of the information sheets. The Ethics Committee of Tarbiat Modares University (Tehran, Iran) approved the study (IR.TMU.REC.1394.181). The trial has been registered in the Iranian Registry of Clinical Trials (IRCT2015090523897N1).

Out of the 82 patients initially recruited, 70 individuals (37 women in the ginger group and 33 women in the placebo group) completed the study. In the ginger group, four women were excluded because of change in the type of medication. Also in the placebo group, two women because of sensitivity and six women because of change in the type of medication (insulin therapy) were excluded from the study.

The inclusion criteria were: singleton pregnancy with normal FBS in the first visit of prenatal care and impaired 2-h oral glucose test at 24–28 weeks of pregnancy.

The exclusion criteria were: using any anti-diabetic drug, afflicted with any diseases such as cardiovascular, respiratory, etc., and being candidate for insulin therapy.

The included patients were randomly assigned to ginger and placebo groups. Ginger tablets or placebo were made by Dineh Company (Tehran, Iran). All women with GDM were referred to the same hospital^’^s Diet and Nutrition Clinic.

The patients received a dose of 1500 mg including three tablets of ginger or placebo daily in three meals (after breakfast, lunch, and dinner) with their dietary regimen order for 6 weeks.

After 6 weeks, the patients were refered again to the same Lab to measure fast insulin, FBS and BS2hpp.

The exclusion criteria included a requirement to treatment with insulin and any sensitivity to ginger or placebo tablets. Confounding variables included taking any anti-diabetic drug (that was controlled by eliminating them), and following dietary regimen for controlling the blood glucose level (which was controlled with matching).

At the first visit, a detailed history including demographic data and history of obstetrics was obtained. Demographic questionnaire was completed by the researcher in which the pregnant women were asked about their address, phone number, date of birth, weight, height, BMI, education level, occupation, education level of husband, gestational age in the time of study, use of any supplementation, and exercise. After completing the questionnaire and giving written consent, they were refered to the Lab of Arash Hospital to measure fast insulin and BS2hpp. HOMA index was calculated using the basic information of FBS and insulin level:

$$ HOMA- IR= fasting\ insulin\ \left( microU/ ml\right)\times fasting\ glucose\ \left( mg/ dl\right)\div 405 $$

Anthropometric measurements were performed at study baseline by the researcher at the Maternity Clinic. Body weight was measured to the nearest 0.1 kg after overnight fasting, without shoes and wearing minimal clothing, by the use of a digital scale (Seca). Height was measured to the nearest 0.1 cm by using a nonstretched tape measure (Seca). BMI was calculated as below:

$$ BMI= weight\ (kg)\div hight\ \left({m}^2\right) $$

Laboratory tests were carried out based on routine standard protocols. Intravenous blood samples were taken in a 10–12 h fasting state and also 2 h post-prandial at the beginning, during the six weeks of the intervention weekly, and at the end of the study by a lab technician. Serum samples were obtained by high-speed (3000 rpm during 15 min) centrifugation. They were frozen immediately at -70^°^C until assay.

The concentration of FBS and BS2hpp was measured by glucose oxidase assay protocol and the enzymatic method. Fasting insulin concentration was measured with the ELISA method by laboratory kit. Resistance to insulin was determined with the homeostasis model formula Homeostasis Model Assessment (HOMA)-IN2.

### Statistical analysis

All statistical analyses were performed by the SPSS software (ver. 22.0) (SPSS Inc., Chicago, IL, USA). The data of normality in distribution were examined by one sample K-S. Group comparisons were carried out with Student’s t-test and Chi-square test. *P* < 0.05 was considered statistically significant.

Since ^“^insulin changes^”^ was one of our outcomes, in order to avoid of making bias, we did not enter and analyze the data of those pregnant women who began insulin therapy during the study.

## Results

There was no significant difference between the ginger and placebo groups regarding the distribution of age, gestational age, Body Mass Index (BMI), housing situation, educational status, physical activity, daily dietary intake and occupation status at the beginning and end of the study (*p*>0.05) (Table 1).

**Table 1:** Basic characteristics of the studied patients

Adv Pharm Bull	Adv Pharm Bull	Adv Pharm Bull	Adv Pharm Bull
Age (years)*	29.68 ± 5.05	31.15 ± 5.26	0.23
BMI*	29.60 ± 3.6	29.50 ± 4.30	0.91
Gestational age)weeks)*	27.72 ± 3.6	27.78 ± 3.60	0.94
Occupation **			
**Housewife**	32 (86.50%)	31 (93.90%)	
**Employer**	5 (13.50%)	2 (6.10%)	0.29
Educational status**			
**High school**	9 (24.50%)	11 (33.30%)	
**Diploma**	19 (54.10%)	13 (39.40%)	0.57
**Bachelor & higher**	9 (24.30%)	9 (27.30%)	
Physical activity**			0.16
**Have exercise**	14 (37.80%)	18 (54.50%)	
**No exercise**	23 (62.20%)	15 (45.50%)	

*Student^,^s T-test (mean ± SD),** Chi-square (n: %)

BMI; Body Mass Index

Table 2 shows that there are no statistically significant differences in the baseline levels of FBS, BS2hpp, serum insulin and HOMA index between the ginger and placebo groups (*p*>0.05) (Table 2).

**Table 2:** Variables before and after intervention

variables	Ginger Mean ± SD	Placebo Mean ± SD	*P*-value**
BS2hpp (before)	127.27 ± 28.40	122.39 ± 27.40	**0.46**
BS2hpp (after)	116.46 ± 21.86	119.58 ± 20.98	**0.54**
*P*-value*	*P* = 0.003	*P* = 0.469	
FBS (before)	96.28 ± 6.24	97.57 ± 3.61	**0.38**
FBS (after)	92.37 ± 7.69	95.78 ± 5.61	**0.04**
*P*-value*	*P* = 0.004	*P* = 0.092	
Insulin (before)	11.60 ± 3.30	11.40 ± 4.50	**0.81**
Insulin (after)	10.03 ± 2.60	11.90 ± 3.70	**0.01**
*P*-value*	*P*<0.001	*P* = 0.095	
HOMA (before)	2.76 ± 0.86	2.74 ± 1.01	**0.92**
HOMA (after)	2.28 ± 0.63	2.82 ± 0.91	**0.05**
*P*-value*	*P*<0.001	*P* = 0.359	

BS2hpp; Blood Sugar 2 h post-prandial

** Student^,^s T-test was carried out for comparison of the groups

*Paired T-test was carried out for comparison of changes between the groups

Ginger treatment significantly reduced the levels of FBS, serum insulin and HOMA index comparing to the placebo group but there was no significant difference between the two groups in BS2hpp after the treatment by the ginger tablets (Table 2).

BS2hpp was reduced in both groups that can be the result of the same dietary regimen given to both groups. BS2hpp reduction difference between the two groups was not statically significant (*p*>0.05) (Table 2).

At last, we used paired T-test to compare means between the study groups (before-after) and analysed the data. According to Table 2, the means of BS2hpp (*P* = 0.003), FBS (*P* = 0.004), HOMA index (*P*<0.001) and serum insulin (*P*<0.001) are reduced significantly in the ginger group (before-after) .

## Discussion

This study was conducted to investigate the effect of ginger on the blood glucose level of women with GDM. The ginger tablets could reduce the level of serum insulin, FBS, and HOMA index but they could not reduce the level of BS2hpp.

The findings of this study showed that treatment with ginger for six weeks could improve glucose status in women with GDM.

All of our variables were improved at the end of the study in comparison to the beginning of intervention. GDM is associated with several advers pregnancy outcomes. The current study showed that this herbal medicine can improve blood glucose status and result in ameliorated outcomes of pregnancy. For example, decreasing in FBS could reduce the number of women who needed to commence insulin therapy; this can be an improved outcome of pregnancy.

Ojewole [6] showed that oral administration of an alcoholic extract of ginger could significantly reduce FBS after 1 h in Streptozotocin (STZ)-diabetic rats. Sanjay P. Akhani et al. [12] reported an increase in insulin levels and a decrease in FBS by treatment with ginger juice. Jafri et al. [8] showed that oral intake of ginger extract in Alloxan-diabetic rats caused a reduction in their blood glucose level. Arabloo [9] found that daily administration of oral ginger in T2D patients caused a reduction in FBS, hemoglobin A1C (HbA1C), HOMA, and insulin level compared with the placebo group. Shidfar et al. [7] also reported a significant improvement in glycemic indices by administration of ginger. Also the study conducted by Shirdel et al. [14] revealed that ginger treatment of diabetic mice could significantly reduce the plasma glucose level, which is clearly in line with our results. Singh et al. [15] found that treatment of mice with 6-gingerols for 12 days could lower FBS and improve other glycemic indices. Our findings are in agreement with these results.

Also Abdulrazaq et al. [16] reported that daily supplementation of diet with aqueous ginger during one month in STZ-diabetic rats resulted in a good reduction in their plasma glucose level.

Mahluji [17] did not report any reduction in the FBS of T2D patients after 2-month administration of 2 g ginger powder daily. This may result from the smaller sample size of their study; the other cause can be the condition of being obese in the inclusion criteria.

To the best of our knowledge, this is the first study that investigates the effect of ginger on BS2hpp in women with GDM. So, for having better results to confirm a reduction in BS2hpp, other studies with larger groups or longer duration of ginger consumption and an elevated dose of ginger should be done.

As reported earlier, our results of insulin changes are in line with the results of previous researchers. GDM is very similar to T2D in pathophysiology so that insufficiency of insulin or resistance to it is problematic. Hence, the favorable results of our study with those of the above studies were expected. Finally, our findings support that ginger can be used in particular groups with GDM; however, more studies with larger sample size and different doses of ginger are required.

Rani et al. [18] mentioned that ginger can inhibit glucosidase, amylase (two key enzymes relevant to T2D and thus improve diabetes. Li [19] suggested that ginger extract containing gingerols increased the uptake of glucose significantly in the rat muscle cells. Another mechanism causing a reduction in the blood glucose levels by ginger is inhibiting the level of hepatic enzyme. This, in turn, prevents the breakdown of hepatic glycogen storage and elevates the activity of enzymes that improve glycogen synthesis. Also a herbal drug with similar effects is nonsteroidal anti-inflammatory drugs (NSAIDs), which can improve the pathways activated with chronic inflammations caused by the diseases such as diabetes [20].

This is the first study that investigated ginger tablets’ effect on the glycemic status and insulin resistance of women with GDM. However, it had some limitations as well. First, some patients were excluded from the intervention group. Moreover, elevated blood glucose level with no need to insulin caused further reduction in the number of eligible patients for the study.

## Conclusions

In conclusion this study results showed that ginger can be usefull in lowering the glucose of women with GDM. So maybe because of safety of this herbal medicien it can be part of our treatments in future.

Finally, conducting similar studies with big sample size and longer treatment and follow-up for better observation of the effect of ginger tablets on improving the status of pateints with GDM is suggested.

## Data Availability

The datasets used and/or analysed during the current study are available from the corresponding author on reasonable request.

## Abbreviations

GDM: Gestational Diabetes Mellitus
DM: Diabetes Mellitus
T2D: Type 2 Diabetes
GTT: Glucose Tolerance Test
FBS: Fast Blood Sugar
HOMA: Homeostasis Model Assessment
BMI: Body Mass Index
BS2hpp: Blood Sugar 2 h post-prandial
STZ: Streptozotocin
HbA1C: Hemoglobin A1C
CAD: Coronary Artery Disease
NSAIDs: Nonsteroidal Anti-Inflammatory Drugs

## References

[1] Clive JP (2010). Gestational diabetes: risk factors and recent advances in its genetics and treatment. Br J Nutr.

[2] Ali AD, Mehrass AA-KO, Al-Adhroey AH, Al-Shammakh AA, Amran AA (2016). Prevalence and risk factors of gestational diabetes mellitus in Yemen. Int J Women's Health.

[3] Mirzamoradi M, Heidar Z, Faalpoor Z, Naeiji Z, JAMAL R (2015). Comparison of glyburide and insulin in women with gestational diabetes mellitus and associated perinatal outcome: a randomized clinical trial.

[4] Wendland EM, Torloni MR, Falavigna M, Trujillo J, Dode MA, Campos MA (2012). Gestational diabetes and pregnancy outcomes-a systematic review of the World Health Organization (WHO) and the International Association of Diabetes in pregnancy study groups (IADPSG) diagnostic criteria. BMC Pregnancy and Childbirth.

[5] Daily JW, Yang M, Kim DS, Park S (2015). Efficacy of ginger for treating type 2 diabetes: a systematic review and meta-analysis of randomized clinical trials. J Ethn Foods.

[6] Ojewole JA (2006). Analgesic, antiinflammatory and hypoglycaemic effects of ethanol extract of Zingiber officinale (roscoe) rhizomes (Zingiberaceae) in mice and rats. Phytother Res.

[7] Shidfar F, Rajab A, Rahideh T, Khandouzi N, Hosseini S, Shidfar S (2015). The effect of ginger (Zingiber officinale) on glycemic markers in patients with type 2 diabetes. J Complement Integr Med.

[8] Jafri SA, Abass S, Qasim M (2011). Hypoglycemic effect of ginger (Zingiber officinale) in alloxan induced diabetic rats (Rattus norvagicus). Pak Vet J.

[9] Arablou T, Aryaeian N, Valizadeh M, Sharifi F, Hosseini A, Djalali M (2014). The effect of ginger consumption on glycemic status, lipid profile and some inflammatory markers in patients with type 2 diabetes mellitus. Int J Food Sci Nutr.

[10] Afshari AT, Shirpoor A, Farshid A, Saadatian R, Rasmi Y, Saboory E (2007). The effect of ginger on diabetic nephropathy, plasma antioxidant capacity and lipid peroxidation in rats. Food Chem.

[11] Aoasa A (2010). Inhibitory effects of aqueous extract of two varieties of ginger on some key enzymes linked to type-2 diabetes in vitro. J Food Nutri Res.

[12] Akhani SP, Vishwakarma SL, Goyal RK (2004). Anti-diabetic activity of Zingiber officinale in streptozotocin-induced type I diabetic rats. J Pharm Pharmacol.

[13] Metzger B, Gabbe S, Persson B, Buchanan T, Catalano P, Damm P (2010). International Association of Diabetes and Pregnancy Study Groups Consensus Panel. International association of diabetes and pregnancy study groups recommendations on the diagnosis and classification of hyperglycemia in pregnancy. Diabetes Care.

[14] Shirdel Z, Mirbalad Zade R, Madani H (2009). Effect of anti diabetic and anti lipidemic of ginger in diabetic rats for aloxan mono hidrate and compare with gliben clamid. Iran J Diabetes lipid Disorders.

[15] Singh AB, Singh N, Maurya R, Srivastava AK (2009). Anti-hyperglycaemic, lipid lowering and anti-oxidant properties of [6]-gingerol in db/db mice. Int. J. Med. Med. Sci.

[16] Abdulrazaq NB, Cho MM, Win NN, Zaman R, Rahman MT (2012). Beneficial effects of ginger (Zingiber officinale) on carbohydrate metabolism in streptozotocin-induced diabetic rats. Br J Nutr.

[17] Mahluji S, Ostadrahimi A, Mobasseri M, Attari VE, Payahoo L (2013). Anti-inflammatory effects of Zingiber Officinale in type 2 diabetic patients. Adv Pharm Bull.

[18] Priya Rani M, Padmakumari K, Sankarikutty B, Lijo Cherian O, Nisha V, Raghu K (2011). Inhibitory potential of ginger extracts against enzymes linked to type 2 diabetes, inflammation and induced oxidative stress. Int J Food Sci Nutr.

[19] Li Y, Tran VH, Duke CC, Roufogalis BD (2012). Gingerols of Zingiber officinale enhance glucose uptake by increasing cell surface GLUT4 in cultured L6 myotubes. Planta Med.

[20] Grzanna R, Lindmark L, Frondoza CG (2005). Ginger—an herbal medicinal product with broad anti-inflammatory actions. J Med Foods.

